# Supplementary material for: The genome of the oomycete Peronosclerospora sorghi, a cosmopolitan pathogen of maize and sorghum, is inflated with dispersed pseudogenes
Source: G3 (Bethesda). 2023 Jan 2;13(3):jkac340. doi: 10.1093/g3journal/jkac340 (PMC9997571; doi:10.1093/g3journal/jkac340)
Supplement: jkac340_Supplementary_Data [file jkac340_supplementary_data.zip › Supplemental_Material_Legends_G3-2022-403616.docx]

**Supplementary Information**

**Table S1. Intermediate assembly statistics.**

**Table S2. RNAseq Read count of *P. sorghi* genes used for differential gene expression analysis.**

**Table S3. Ranked count of Pfam domains encoded by distinct assembled transcripts.**

**Table S4. List of oomycete assemblies used for orthology analysis.**

**Table S5. Ranked count of Pfam domains encoded by distinct assembled transcripts that do not overlap annotated genes.**

**Table S6. Ranked count of Pfam domains encoded by distinct assembled transcripts that overlap annotated genes.**

**Table S7. Pfam domains encoded by *P. sorghi* genes identified as differentially regulated**

**File S1. Metagenomic classification of unassigned, assembled transcripts.** Interactive HTML produced using Krona tools. Very few of the unassigned transcripts were assigned to the Oomycota.

**File S2. Maximum likelihood tree calculated by RAxML**. Newick format file used to generate Fig. 2.
